# Patient Health Questionnaire‐9 predicts the functional outcome of stroke patients in convalescent rehabilitation ward

**DOI:** 10.1002/brb3.1856

**Published:** 2020-09-20

**Authors:** Masahiro Nakamori, Eiji Imamura, Keisuke Tachiyama, Teppei Kamimura, Yuki Hayashi, Hayato Matsushima, Hiroyuki Okamoto, Tatsuya Mizoue, Shinichi Wakabayashi

**Affiliations:** ^1^ Department of Neurology Suiseikai Kajikawa Hospital Hiroshima Japan; ^2^ Department of Clinical Neuroscience and Therapeutics Hiroshima University Graduate School of Biomedical and Health Sciences Hiroshima Japan; ^3^ Department of Rehabilitation Suiseikai Kajikawa Hospital Hiroshima Japan; ^4^ Department of Neurosurgery Suiseikai Kajikawa Hospital Hiroshima Japan

**Keywords:** activities of daily living, depression, patient health questionnaire, stroke rehabilitation

## Abstract

**Introduction:**

Poststroke depression (PSD) negatively affects the functional outcome of stroke patients. Patient Health Questionnaire‐9 (PHQ‐9) is a validated screening tool for detecting PSD. This study investigated the relationship between PHQ‐9 score and functional outcomes in stroke patients in a convalescent rehabilitation ward by evaluating functional independence measure (FIM) gain scores and the proportion of patients discharged.

**Methods:**

In this retrospective study conducted from January 2017 to September 2019, consecutive stroke patients who were admitted to the convalescent rehabilitation ward and could answer PHQ‐9 were assessed. The association between PHQ‐9 scores at the time of admission to the convalescent rehabilitation ward and outcomes (FIM gain score and the proportion of patients discharged) was statistically analyzed.

**Results:**

Among the 215 patients enrolled in the study, 62 (28.8%) were assessed as having depression, in whom PHQ‐9 scores were 5 or above. Multivariate analysis revealed that the PHQ‐9 score on admission to the convalescent rehabilitation ward was a significant independent factor influencing the FIM gain score (*p* = .009). In addition, a multivariate analysis revealed that the PHQ‐9 score at the time of admission to the convalescent rehabilitation ward was a significant independent factor influencing the inability to discharge a patient (odds ratio 1.24, 95% confidence interval 1.12–1.39, *p* < .001).

**Conclusions:**

The PHQ‐9 score is a useful tool for predicting patient functional outcome, admission to the facility, and screening for poststroke depression.

## Graphical abstract

PHQ‐9 score on admission to convalescent rehabilitation ward was an independent significant factor influencing the FIM gain score and the inability to discharge home.

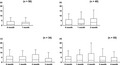

## 1 INTRODUCTION

The association between psychiatric disorders and cerebrovascular diseases was described many years ago (Robinson & Jorge, 2016). Among them, poststroke depression (PSD) is a representative psychiatric disorder. Depression is quite common in patients with stroke compared with that in patients with physical impairment of similar extent (Folstein et al., 1977). Approximately one‐third of patients are affected by PSD, making it a serious social and public health problem; therefore, antidepressant preventive and curative therapies are worth investigating (Babkair, 2017; Hackett et al., 2018; Pohjasvaara et al., 1998; Villa et al., 2018). Further, depression negatively affects the functional outcome of stroke patients, response to rehabilitation, and quality of life (Ramasubbu et al., 1998; Starkstein et al., 1988).

Randomized controlled trials for the prevention of PSD have shown that antidepressants significantly decreased its incidence compared to placebos (Lipsey et al., 1984). Early intervention with antidepressants appears to enhance physical recovery from stroke and increase survival for up to 10 years (Robinson & Jorge, 2016).

While depression is only diagnosed via a clinical interview, there are several scales for evaluating depressive symptom burden including but not limited to the Beck Depression Inventory, Wechsler Depression Rating Scale, and the Quick Inventory of Depressive Symptomatology. The Patient Health Questionnaire is a simple and self‐answered questionnaire which was developed for primary care physicians (Spitzer et al., 1999). The Patient Health Questionnaire (PHQ‐9) is a nine‐item questionnaire, based on major depressive disorder symptoms, designed to screen and evaluate the severity of depression in primary care and other medical settings (Kroenke et al., 2001; Levis et al., 2019). It is simple and useful for repetitive evaluations. A previous meta‐analysis has validated the accuracy of PHQ‐9 to screen for depression (Levis et al., 2019). Moreover, it was reported that PHQ‐9 is valid and useful for detecting PSD (Prisnie et al., 2016; Trotter et al., 2019). Patient Health Questionnaire is becoming popular for evaluating PSD. With respect to stroke treatment and especially rehabilitation, the ultimate purpose of evaluation for depression is to improve patient outcomes. It has been reported that the Geriatric Depression Scale (GDS) was associated with Functional Independence Measure (FIM) gain scores (Tsuchiya et al., 2016). Originally GDS consists of 30 items, whereas PHQ‐9 has fewer questions and nine essential items from the Diagnostic and Statistical Manual of Mental Disorders, Fifth edition. The patients may be burdened while answering many questions. For this reason, simplicity and reliability are expected with PHQ‐9. However, so far, none of the studies have investigated the association between PHQ‐9 score and patient outcome.

The Japanese national insurance system introduced a convalescent rehabilitation ward in the year 2000. Maximal coverage included therapy sessions for 3 hr a day, 7 days a week. Patients who need assistance after their acute phase treatment were transferred to a convalescent rehabilitation ward. In Japan, these wards contribute significantly to improving patient outcomes (Miyai et al., 2011). According to the Japanese national insurance system, patients must be admitted to these wards within 60 days of the onset of stroke, and the maximum stay is limited to 150 days. Thus, the prediction of functional outcomes is essential in the postacute phase to ensure appropriate post‐treatment planning.

In this study, we investigated the relationship between PHQ‐9 and functional outcomes for stroke patients in a convalescent rehabilitation ward by evaluating FIM gain scores and the proportion of patients who were admitted to this facility.

## 2 MATERIALS AND METHODS

### 2.1 Subjects

This retrospective study was conducted at the Suiseikai Kajikawa Hospital, Hiroshima, Japan. During the period from 1 January 2017 to 30 September 2019, consecutive stroke patients who were admitted to the convalescent rehabilitation ward and who could answer the PHQ‐9 were enrolled in the study. Patients who had been diagnosed with depression before the onset of stroke and those who had a recurrence of stroke during hospitalization were excluded. The study protocols were approved by the ethics committee of Suiseikai Kajikawa Hospital and were performed in accordance with national government guidelines based on the 1964 Declaration of Helsinki. The requirement for informed consent for this study was waived owing to its retrospective nature. At the time of admission, the included patients had agreed that their data could be used for future studies.

### 2.2 Data acquisition

Two researchers (KT and YH) collected the demographic and clinical characteristics of the patients, including the age at admission, sex, whether or not the patient was single, past history (cerebrovascular disease, hypertension, diabetes mellitus, dyslipidemia, atrial fibrillation), scores on admission to the convalescent rehabilitation ward [PHQ‐9 score, Mini‐Mental State Examination (MMSE) score, FIM score], and the duration of stay in the convalescent rehabilitation ward. Stroke subtypes were classified by two stroke neurologists (MN and EI) according to the Trial of Org 10,172 in the Acute Stroke Treatment classification (Adams et al., 1993). Additionally, stroke lesions were identified using magnetic resonance imaging. Neurological deficits were estimated using the National Institutes of Health Stroke Scale scoring system (Lyden et al., 1994). Both PHQ‐9 and FIM scores were evaluated every month from the time of admission to discharge. The FIM gain score was calculated as the difference in scores between the first and last FIM score. Hypertension was defined as the use of antihypertensive medication before admission or a confirmed blood pressure of ≥140/90 mm Hg at rest measured 2 weeks after the onset of stroke. Diabetes mellitus was defined as a glycated hemoglobin level ≥ 6.5%, fasting blood glucose level ≥ 126 mg/dl, or use of antidiabetes medication. Dyslipidemia was defined as total cholesterol level ≥ 220 mg/dl, low‐density lipoprotein cholesterol level ≥ 140 mg/dl, high‐density lipoprotein cholesterol level < 40 mg/dl, triglyceride levels ≥ 150 mg/dl, or use of antihyperlipidemia medication. Atrial fibrillation was defined as follows: (a) a history of sustained or paroxysmal atrial fibrillation, or (b) atrial fibrillation detection on arrival or during admission.

### 2.3 PHQ‐9 score

PHQ‐9 is a nine‐item questionnaire completed by the patient. It consists of nine questions [1. Little interest or pleasure in doing things? 2. Feeling down, depressed, or hopeless? 3. Trouble falling or staying asleep, or sleeping too much? 4. Feeling tired or having little energy? 5. Poor appetite or overeating? 6. Feeling bad about yourself ‐ or that you are a failure or have let yourself or your family down? 7. Trouble concentrating on things, such as reading the newspaper or watching television? 8. Moving or speaking so slowly that other people could have noticed? or the opposite—being so fidgety or restless that you have been moving around a lot more than usual? 9. Thoughts that you would be better off dead, or of hurting yourself in some way?]. Each question is scored from 0 to 3 points according to the frequency in the preceding 2 weeks, and a higher score reflects poorer condition. A score of 10 points or higher reflects major depression, and a score of 5 points or higher reflects mild depression.^11^ PHQ‐9 scores were evaluated every month from the time of admission to discharge.

### 2.4 Statistical analysis

Data are expressed as mean ± standard deviation or the median [25% indicates interquartile range (IQR)–75% IQR] for continuous variables, and frequencies and percentages for discrete variables. Statistical analysis was performed using the JMP 14.0 statistical software (SAS Institute Inc., Cary, NC, USA). The statistical significance of intergroup differences was assessed using an unpaired *t* test or Mann–Whitney *U* test or analysis of variance (for continuous variables) or the Fisher exact test or chi‐square test (for discrete variables), as appropriate. Baseline patient data were analyzed, and two‐step strategies were employed to assess the relative importance of variables and their association with FIM gain scores, home discharge, and PHQ‐9 scores using a least square linear regression analysis or multiple logistic analysis. We first performed a univariate analysis with *p* < .10, followed by a multifactorial analysis with selected factors. If there were many selected factors, a bidirectional stepwise regression analysis was used to select factors and *p* = .20 was used as a cutoff level. We considered *p* < .05 to reflect statistical significance.

## 3 RESULTS

During the study period, 258 stroke patients were admitted to the convalescent rehabilitation ward. Twenty‐six patients could not answer the PHQ‐9 due to altered consciousness or refusal. Five patients had been diagnosed with depression prior to the onset of stroke. There was a reoccurrence of stroke in 12 patients during hospitalization. Therefore, 215 patients were included in this study after excluding the above. The background and characteristics of the subjects at the time of enrollment are shown in Table 1. The median score of PHQ‐9 during their admission to the convalescent rehabilitation ward was 2 (0, 5). There were 21 (9.8%) patients whose PHQ‐9 scores were 10 or higher, which was regarded as major depression. There were 62 (28.8%) patients whose scores were 5 or higher, which was regarded as minor depression.

**TABLE 1:** Patient characteristics

	*n* = 215
Age, year	72.0 ± 12.9
Sex (female), *n* (%)	99 (46.0)
Solitude, *n* (%)	45 (20.9)
History	
Cerebrovascular disease, *n* (%)	64 (29.8)
Hypertension, *n* (%)	177 (82.3)
Diabetes mellitus, *n* (%)	52 (24.2)
Dyslipidemia, *n* (%)	123 (57.2)
Atrial fibrillation, *n* (%)	43 (20.0)
NIHSS score, median (minimum–maximum)	5 (0, 27)
Stroke subtypes	
Large‐artery atherosclerosis, *n* (%)	38 (17.7)
Cardioembolism, *n* (%)	32 (14.9)
Small‐vessel occlusion, *n* (%)	16 (7.4)
Others, *n* (%)	71 (33.0)
Intracranial hemorrhage, *n* (%)	58 (27.0)
Stroke lesion	
Side of lesion	
Left, *n* (%)	111 (51.6)
Supratentorial, *n* (%)	179 (83.3)
Cerebral cortex	
Frontal lobe, *n* (%)	55 (25.6)
Temporal lobe, *n* (%)	33 (15.3)
Parietal lobe, *n* (%)	29 (13.5)
Occipital lobe, *n* (%)	17 (7.9)
Insular cortex, *n* (%)	21 (9.8)
Subcortical	
Corona radiata, *n* (%)	91 (42.3)
Basal ganglia, *n* (%)	80 (37.2)
Capsulae internae, *n* (%)	37 (17.2)
Thalamus, *n* (%)	28 (13.0)
Infratentorial	
Brain stem, *n* (%)	27 (12.6)
Cerebellum, *n* (%)	17 (7.9)
Scores at the entrance of convalescent rehabilitation wards	
PHQ‐9 score, median (minimum–maximum)	2 (0, 22)
MMSE score, median (minimum–maximum)	25 (6, 30)
FIM score, median (minimum–maximum)	71 (18, 123)
Usage of antidepressant drug, *n* (%)	15 (7.0)
Staying convalescent rehabilitation wards, days	82.0 ± 43.0
FIM gain score, median (minimum–maximum)	22 (−13, 76)
Home discharge, *n* (%)	168 (78.1)

Note

Abbreviations: FIM, Functional Independence Measure; MMSE, Mini‐Mental State Examination; NIHSS, National Institutes of Health Stroke Scale; PHQ‐9, Patient Health Questionnaire‐9.

Data are presented as the mean ± standard deviation, median [25% indicates interquartile range (IQR)–75% IQR], or number of patients (%).

The median of the FIM gain scores was 22 (9, 31). We analyzed the association with FIM gain score using the factors listed in Table 1. A univariate analysis revealed that age, a history of cerebrovascular disease and atrial fibrillation, stroke subtypes, stroke lesion in the temporal lobe, insular cortex, and corona radiata, PHQ‐9 score, MMSE score, and FIM score during admission to the convalescent rehabilitation ward were associated with FIM gain scores (*p* < .10). Multivariate analysis identified that a history of cerebrovascular disease, atrial fibrillation, stroke subtype (cardioembolism and small‐vessel occlusion), stroke lesion in the corona radiata, PHQ‐9 score, MMSE score, and FIM score at the time of admittance to the convalescent rehabilitation ward were significant independent factors (*p* < .05) (Table 2).

**TABLE 2:** Factors influencing FIM gain scores

	Univariate analysis	Multivariate analysis		
	*p*‐value	Partial regression coefficient	95% CI	*p*‐value
Age, yr	.013	−0.133	−0.320–0.054	.162
Sex (female)	.627			
Solitude	.207			
History				
Cerebrovascular disease	<.001	−4.202	−6.448 to −1.956	<.001
Hypertension	.917			
Diabetes mellitus	.156			
Dyslipidemia	.295			
Atrial fibrillation	.035	−4.018	−7.998 to −0.038	.048
NIHSS score	.364			
Stroke subtypes	.027			
Large‐artery atherosclerosis				
Cardioembolism		7.903	0.077–15.73	.048
Small‐vessel occlusion		−6.807	−12.991 to −0.622	.031
Others				
Intracranial hemorrhage				
Stroke lesion				
Side of lesion				
Left	.552			
Supratentorial	.268			
Cerebral cortex				
Frontal lobe	.705			
Temporal lobe	.092	−0.580	−3.194–4.353	.762
Parietal lobe	.281			
Occipital lobe	.393			
Insular cortex	.091	−0.462	−5.311–6.235	.875
Subcortical				
Corona radiata	.033	−2.608	−5.087 to −0.129	.039
Basal ganglia	.377			
Capsulae internae	.002			
Thalamus	.132			
Infratentorial				
Brain stem	.874			
Cerebellum	.148			
Scores at the time of admission to convalescent rehabilitation ward				
PHQ‐9 score	.040	−0.745	−1.302 to −0.188	.009
MMSE score	.002	1.101	0.705–1.498	<.001
FIM score	.001	−0.449	−0.553 to −0.346	<.001
Usage of antidepressant drug	.727			

Note

Abbreviations: CI, confidence interval; FIM, Functional Independence Measure; MMSE, Mini‐Mental State Examination; NIHSS, National Institutes of Health Stroke Scale; PHQ‐9, Patient Health Questionnaire‐9.

Forty‐seven (21.9%) patients were transferred to institutions such as nursing homes and welfare facility for the elderly. We analyzed the association between any of the factors with patients not being able to be discharged using the factors listed in Table 1. Univariate analysis revealed that age, sex, solitude, a history of cerebrovascular disease, dyslipidemia, atrial fibrillation, National Institutes of Health Stroke Scale (NIHSS) score, stroke subtypes, stroke lesions of the frontal lobe, temporal lobe, parietal lobe, insular cortex, capsulae internae, PHQ‐9 score, MMSE score, and FIM score at the time of admittance to the convalescent rehabilitation ward were associated with the impossibility of discharging the patients home (*p* < .10). Thus, we used a bidirectional stepwise regression analysis and selected atrial fibrillation, PHQ‐9 score, and MMSE score. Multivariate analysis revealed that atrial fibrillation (odds ratio 3.56, 95% confidence interval [CI] 1.34–9.54, *p* = .011), PHQ‐9 score (odds ratio 1.24, 95% CI 1.12–1.39, *p* < .001), and MMSE score (odds ratio 0.85, 95% CI 0.79–0.90, *p* < .001) were independently associated with transfer to institutions such as nursing homes and welfare facilities (Table 3).

**TABLE 3:** Factors influencing patient discharge

	Multivariate analysis		
	Odds ratio	95% CI	*p‐*value
Atrial fibrillation	3.56	1.34–9.54	.011
PHQ‐9 score	1.24	1.12–1.39	<.001
MMSE score	0.85	0.79–0.90	<.001

Note

CI, confidence interval; MMSE, Mini‐Mental State Examination; PHQ‐9, Patient Health Questionnaire‐9.

We investigated the association of PHQ‐9 scores with the factors listed in Table 1. Univariate analysis revealed that a history of atrial fibrillation, NIHSS score, stroke lesions of the parietal lobe, occipital lobe, insular cortex, and basal ganglia, MMSE score, and FIM score at the time of admission to the convalescent rehabilitation ward were associated with PHQ‐9 scores (*p* < .10). Multivariate analysis identified that NIHSS and MMSE score at the time of admission to the convalescent rehabilitation ward were significant independent factors (*p* < .05) (Table 4). The stroke lesions were not associated with PHQ‐9 score in this study.

**TABLE 4:** Factors associated with PHQ‐9 scores

	Univariate analysis	Multivariate analysis		
	*p*‐value	Partial regression coefficient	95% CI	*p*‐value
Age, yr	.790			
Sex (female)	.761			
Solitude	.864			
History				
Cerebrovascular disease	.591			
Hypertension	.770			
Diabetes mellitus	.414			
Dyslipidemia	.634			
Atrial fibrillation	.017	0.275	−1.026–0.476	.471
NIHSS score	<.001	0.112	0.027–0.196	.010
Stroke subtypes	.458			
Large‐artery atherosclerosis				
Cardioembolism				
Small‐vessel occlusion				
Others				
Intracranial hemorrhage				
Stroke lesion				
Side of lesion				
Left	.493			
Supratentorial	.594			
Cerebral cortex				
Frontal lobe	.597			
Temporal lobe	.318			
Parietal lobe	.057	0.050	−1.014–0.914	.919
Occipital lobe	.016	−0.856	−0.080–1.793	.073
Insular cortex	.003	0.004	−1.254–1.246	.995
Subcortical				
Corona radiata	.293			
Basal ganglia	.051	0.132	−0.667–0.403	.627
Capsulae internae	.191			
Thalamus	.542			
Infratentorial				
Brain stem	.915			
Cerebellum	.287			
Scores at the entrance of convalescent rehabilitation wards				
MMSE score	<.001	−0.101	−0.194 to −0.008	.034
FIM score	<.001	−0.020	−0.046–0.006	.139
Usage of antidepressant drug	.492			

Note

CI, confidence interval; FIM, Functional Independence Measure; MMSE, Mini‐Mental State Examination; NIHSS, National Institutes of Health Stroke Scale; PHQ‐9, Patient Health Questionnaire‐9.

**FIGURE 1:**
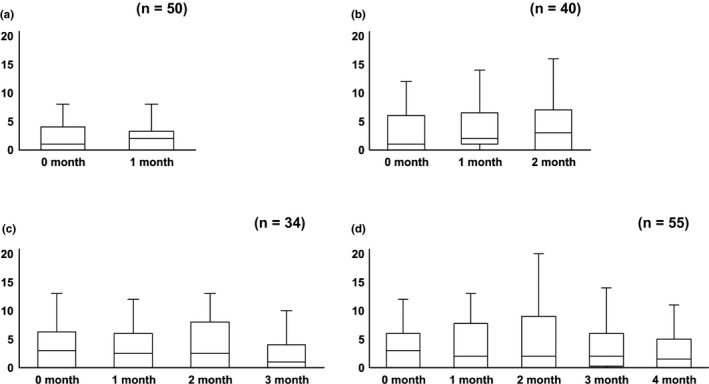
Transitive graphs of PHQ‐9 per month. (a)–(d) shows the transitive PHQ‐9 scores for hospital‐stay periods of 1, 2, 3, and 4 months, respectively. PHQ‐9, Patient Health Questionnaire‐9

Transitive graphs of PHQ‐9 score are shown in Figure 1. The times at which PHQ‐9 scores were evaluated varied because the number of hospital‐stay days differed among patients. Thus, we have separately shown the scores according to the hospital‐stay period. Figure 1(a)–(d) shows the transitive PHQ‐9 scores for hospital‐stay periods 1, 2, 3, and 4 or more months, respectively. In all the graphs, PHQ‐9 score increased after 2 months from the time of admission. However, statistical analyses did not show a significant difference.

Further, we investigated antidepressant drug usage. A total of 15 (6.7%) patients were receiving antidepressant drugs. In this study, there was no association between antidepressant drug usage and background factors such as FIM gain score or the proportion of patients discharged.

## 4 DISCUSSION

We used the PHQ‐9 score to assess poststroke depressive burden in a convalescent rehabilitation ward, which is an original Japanese insurance system. PHQ‐9 scores were independently associated with FIM gain scores and the proportion of patients discharged. PHQ‐9 scores tended to worsen 2 months after admission to the convalescent rehabilitation ward. In this study, PHQ‐9 scores on admission were related to the NIHSS score and MMSE score, but not the stroke lesion. In our study, 28.8% patients had a PHQ‐9 score of 5 points or more, and 9.8% patients had a score of 10 points or more. According to our study, approximately one third of patients might be affected by PSD including mild depression, which was consistent with the results of previous reports (Hackett et al., 2018; Pohjasvaara et al., 1998).

PHQ‐9 scores at the time of admission to the convalescent rehabilitation ward were independently associated with FIM gain scores. There are many reports in which PSD worsened the response to rehabilitation and the functional outcome of patients (Ramasubbu et al., 1998; Starkstein et al., 1988). In Japan, GDS was associated with FIM gain scores in convalescent rehabilitation wards (Tsuchiya et al., 2016). Our results are consistent with those of previous reports, though this is the first study to investigate the relationship between PHQ‐9 and FIM gain scores. PHQ‐9 scores are also a useful predictor for functional outcomes in convalescent rehabilitation wards.

In addition, we investigated the relationship between PHQ‐9 and the proportion of patients who were admitted to the facility. Solitude, PHQ‐9 score, MMSE score, and FIM score at the time of admittance to the convalescent rehabilitation ward were significant independent factors for admission to a facility. Home discharge is an indication for the outcome of stroke rehabilitation. The early evaluation and appropriate intervention including rehabilitation plans and setting up of goals will promote home discharge and improve the medical economy. In this study, PHQ‐9 scores were independently associated with the proportion of patients discharged, regardless of social background. PHQ‐9 scores may be an important factor for the prediction and intervention for home discharge.

We evaluated the association between PHQ‐9 and stroke lesion. There are many reports on constitutional abnormality and PSD. The left frontal lobe has been typically reported as a causative lesion site for PSD (Robinsonet al., 1985; Shimoda & Robinson, 1999). According to a meta‐analysis, PSD in the acute stroke phase is significantly associated with frontal and basal ganglia lesions (Douven et al., 2017). However, many controversial results have been recorded (Carson et al., 2000; Dam et al., 1989; House et al., 1990; Kim & Choi‐Kwon, 2000; Singh et al., 1998; Sinyor et al., 1986), in which there was no association between stroke lesion and PSD. We compared both sides of the brain, the supra/infratentorial, and each lobe; however, no associations were discovered. One reason might be patient selection. We focused on the patients admitted to the convalescent rehabilitation ward and did not include all stroke patients admitted to our hospital. To investigate the relationship between stroke lesion and PSD, stroke patients need to be included on a wider basis and stratified comparison should be performed in detail.

The pathophysiology of PSD is presumably multifactorial, involving a combination of various mechanical dysfunctions in the context of psychosocial distress. Some progress has been made in understanding the pathophysiology of PSD. Many risk factors of PSD have been reported, including but not limited to inflammatory cytokines (Jiao et al., 2016), genetic and epigenetic variations (Kim, et al., 2013; Kim, et al., 2013; Kohen et al., 2008), and white matter disease (Krishnan et al., 1997). Further elucidation of the mechanism of PSD may ultimately lead to specific targeted treatments (Robinson & Jorge, 2016).

It is important to detect PSD and start therapeutic interventions earlier on (Volz et al., 2016). Antidepressants significantly reduce the incidence of PSD and early antidepressant treatment of PSD appears to enhance both physical recovery and recovery from stroke (Lipsey et al., 1984; Robinson & Jorge, 2016). In this retrospective study, only 7.0% of the patients received antidepressant drugs, and there was no association between antidepressant drug use and FIM gain scores or the proportion of patients discharged. The reason was that the number of patients who received antidepressant drugs was small and as this study was not randomized it might include selection bias.

This study had several limitations. First, the study was conducted in a single institution and thus may be biased by the single‐center effect and clustering of observations. Future multicenter collaborative research is needed to eliminate such effects. Second, we did not assess patients using other depression evaluation scales or compare the results with them. A detailed evaluation may contribute to understanding the character of PSD and enable medical staff to better implement a suitable intervention. Third, we could not evaluate the effect of antidepressant drugs adequately. This was a retrospective study, and the number of patients who received antidepressant drugs remained small. However, large nonrandomized studies have already been conducted, which revealed the effectiveness of antidepressant therapy. It is important to detect PSD earlier and begin an intervention. Based on this, PHQ‐9 is a simple, easy, and repetitive evaluation method for the detection of PSD and prediction of functional outcomes.

## 5 CONCLUSIONS

Poststroke depression worsens the FIM gain score and prevents the discharge of patients from convalescent rehabilitation wards. The PHQ‐9 score is a useful tool for predicting patient outcomes as well as screening for poststroke depression.

## CONFLICT OF INTEREST

The authors have stated explicitly that there are no conflicts of interest in connection with this article.

## AUTHORS' CONTRIBUTIONS

Masahiro Nakamori, Eiji Imamura, Hiroyuki Okamoto, Tatsuya Mizoue, and Shinichi Wakabayashi conceived and designed the experiments. Eiji Imamura, Keisuke Tachiyama, Yuki Hayashi, and Hiroyuki Okamoto performed the experiments. Masahiro Nakamori, Teppei Kamimura, and Hayato Matsushima analyzed the data. Masahiro Nakamori, Hiroyuki Okamoto, and Tatsuya Mizoue contributed reagents/materials/analysis tools. Masahiro Nakamori, Eiji Imamura, and Shinichi Wakabayashi wrote the paper.

### Peer Review

The peer review history for this article is available at https://publons.com/publon/10.1002/brb3.1856.

## Data Availability

The data that support the findings of this study are available from the corresponding author on reasonable request.
